# Needle-type organic electrochemical transistor for spatially resolved detection of dopamine

**DOI:** 10.1007/s00604-020-04352-1

**Published:** 2020-06-09

**Authors:** Federica Mariani, Thomas Quast, Corina Andronescu, Isacco Gualandi, Beatrice Fraboni, Domenica Tonelli, Erika Scavetta, Wolfgang Schuhmann

**Affiliations:** 1 Dipartimento di Chimica Industriale “Toso Montanari”, Università di Bologna, Viale del Risorgimento 4, 40136 Bologna, Italy; 2 Analytical Chemistry-Center for Electrochemical Sciences (CES), Faculty of Chemistry and Biochemistry, Ruhr University Bochum, Universitätsstraße 150, 44780 Bochum, Germany; 3 Chemical Technology III, Faculty of Chemistry and Center for Nanointegration (CENIDE), University Duisburg Essen, Carl-Benz-Str. 201, D-47057 Duisburg, Germany; 4 Dipartimento di Fisica e Astronomia, Università di Bologna, Viale Berti Pichat 6/2, 40127 Bologna, Italy

**Keywords:** Nanoelectrode, PEDOT:PSS, Organic electrochemical transistor, Bioelectronics, Dopamine

## Abstract

In this work, the advantages of carbon nanoelectrodes (CNEs) and orgonic electrochemical transistors (OECTs) were merged to realise nanometre-sized, spearhead OECTs based on single- and double-barrel CNEs functionalised with a conducting polymer film. The needle-type OECT shows a high aspect ratio that allows its precise positioning by means of a macroscopic handle and its size is compatible with single-cell analysis. The device was characterised with respect to its electrolyte-gated behaviour and was employed as electrochemical sensor for the proof-of-concept detection of dopamine (DA) over a wide concentration range (10^−12^—10^−6^ M). Upon application of fixed drain and gate voltages (*V*_*d*_ = − 0.3 V, *V*_*g*_ = − 0.9 V, respectively), the nano-sized needle-type OECT sensor exhibited a linear response in the low pM range and from 0.002 to 7 μM DA, with a detection limit of 1 × 10^−12^ M.

**Electronic supplementary material:**

The online version of this article (10.1007/s00604-020-04352-1) contains supplementary material, which is available to authorized users.

**Figure:**
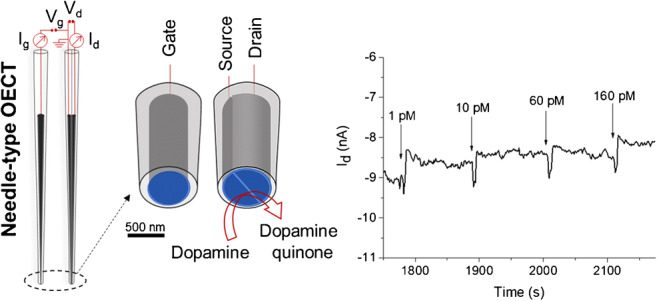
Graphical abstract

## Introduction

The perspective of downscaling a device architecture into the nano range is considered an essential technological evolution in the field of integrated circuits and holds great potential for recording biological signals. In this scenario, the development of miniaturised bioelectronic systems is of particular interest for highly sensitive and resolved detection of crucial biomarkers. Paradigmatic is the case of dopamine (DA), an endogenous catecholamine that exerts a prevalent role in the neural tissues as a neurotransmitter and is involved in the regulation of emotional and cognitive functions, as well as neuroendocrine control [1, 2]. DA concentrations in vivo typically lie in the low nanomolar range [3] and naturally occurring phasic firing of a single dopamine neuron produces variations in DA concentration in a sub-second time scale that are spatially localised in close proximity to the synaptic contact, followed by rapid diffusion and reuptake [4]. Indeed, neurotransmitter signalling pathways comprise a complex cascade of release and binding events to intra and extrasynaptic receptors and secondary processes in the dopaminergic system that can be triggered in response to external stimuli, such as sensory inputs or assumption of psychostimulants [5]. DA transients are enhanced upon administration of drugs of abuse [6], while dysfunctions and neurodegeneration of the dopaminergic system have been correlated with oxidative stress [7] and severe neurological disorders such as Parkinson’s disease [8]. Real-time detection of DA fluctuations in vivo or ex vivo poses several analytical challenges to existing techniques, as a combination of high sensitivity, selectivity and stability with spatial and temporal resolution is required. Among the most commonly used techniques for the measurement of neurotransmitter dynamics, microelectrodes were used directly or coupled to sampling methods such as microdialysis [9]. While microdialysis provides excellent chemical selectivity, a certain volume of dialysate has to be collected, thus lacking the temporal resolution that is required to detect discrete neurochemical events. Moreover, since microdialysis probes have typical diameters of hundreds of μm, information about spatial distribution is partially lost [10]. Contrarily, high sampling rates in the millisecond timescale [11] are obtained using microelectrodes that can be positioned at the desired location to perform local electrochemical sensing by constant-potential amperometry or fast-scan cyclic voltammetry e.g. using carbon fibre microelectrodes (10 μm diameter) [12]. Still at an initial research stage, superior spatial resolution can be obtained by the use of sub-micrometric, needle-type structures, such as carbon nanopipettes (CNPEs) and nanoelectrodes (CNEs), whose high aspect ratio provides a mean of interfacing a nanoscopic structure with a macroscopic handle without a need for any assembly, but allowing precise positioning of the sensing device at the single-cell level [13]. Thanks to the sharp geometry that facilitates penetration and implantation for localised measurements in distinct regions of small organisms, a CNPE sensor has been used to detect endogenous DA release in the dopaminergic centres of *Drosophila larvae* with a limit of detection of 25 nM [14]. Recent advancements towards the reproducible fabrication of CNEs have been achieved, thus significantly improving their manipulation and reliable applicability for analytical purposes [15]. For instance, a syringaldazine-based CNE has been reported as voltammetric nanosensor for pH imaging at high scan rate (0.66 V s^−1^) and with high spatial resolution [16]. However, electrode fouling, background interference and selectivity remain major issues that should be addressed to produce reliable electrochemical sensors [3, 12].

Organic electrochemical transistors (OECTs) are a special class of electronic devices that are gaining momentum in the design of novel bioelectronic interfaces [17, 18]. A thin film of an organic semiconductor (channel) is deposited between individually addressable source (S) and drain (D) electrodes. While the source is grounded, a bias (*V*_*d*_) is applied to the drain electrode that generates a current flowing through the channel (*I*_*d*_) that is collected at the drain. An electrolyte is in direct contact with the channel material and the third electrode, i.e. the gate (G). The transistor channel is typically made of the polymeric salt poly(3,4-ethylenedioxythiophene): poly(styrenesulfonate) (PEDOT:PSS), where the intrinsically conducting polymer PEDOT exhibits biocompatible features [19, 20] and has the capability of transducing ionic fluxes into electronic signals. Indeed, not only the charge is shuttled through both holes and ionic species, via electronic and mass transport, respectively, as in a mixed conductor [21], but also ionic fluxes can induce electronic currents and vice versa, thanks to a strong ionic/electronic coupling that is corroborated by reversible electrochemical doping [22]. When the OECT channel is made of PEDOT:PSS, such a phenomenon is controlled by the application of the gate voltage, *V*_*g*_, through the electrolyte solution. Indeed, owing to the high difference in conductivity between doped and undoped states of conjugated polymers (i.e. PEDOT^+^ and PEDOT, respectively), the polarisation of the gate electrode generates an ionic current (*I*_*g*_) across the medium that is able to induce a pronounced switch between an “on” (conductive) and an “off” (nonconductive) state of the channel [23]. OECT-based sensors have been realised that combine the features of a sensor and an amplifier, where small potential changes due to the analyte lead to a pronounced variation of the output signal, ensuring high sensitivity and improved signal to noise ratio. OECT sensors have been reported for the detection of a variety of biologically relevant analytes with nanomolar and sub-nanomolar detection limits, thus exceeding the performance of conventional electrochemical sensors. These include enzymatic glucose biosensing [24, 25], detection of epinephrine [26], dopamine [27, 28] and ions [29]. Integrated with microfluidic systems, OECT sensors have been used for liposome sensing [30], and monitoring of 3D cell cultures membrane integrity [31]. Also, lactate detection in tumour cell cultures has been recently carried out [32], and an OECT sensor capable of attomolar detection of immunoglobulin G has been reported [33].

Sub-micrometric organic channels allow to reach a very high transistor density within a bioelectronic interface, thus increasing the number of recording sites. Donahue et al. contextualise this topic and report the fabrication and characterisation of OECTs with vertically stacked contacts, alternative to the conventionally planar structure, with minimum channel length of 450 nm [34]. More recently, a channel length of 50 nm has been reached in an ion-gel-gated OECT based on different electrochemically doped polymers [35]. The most appealing feature of downscaled OECTs is the capability to achieve fast operation speed. In this regard, a thorough study has been recently carried out on OECTs with submicrometric channel sizes fabricated on electrode gaps by electromigration-induced break junction technique. Here, the authors report superior amplifying properties of fast varying signals and time responses in the millisecond scale [36]. However, it should be noted that all devices reported so far are based on chip-like geometries.

In this scenario, the combination of a transistor-like configuration with nanometric, needle-type objects represents a clear breakthrough for single-cell analysis and has led to highly sensitive and fast responses, thanks to the transistor amplification. In this view, field-effect transistors (FETs) and nanopore platforms have been merged to develop a nanopipette-based polypyrrole (PPy) ionic-FET. The device was used to detect single-molecule translocation events of DNA and, with insulin-modified PPy, IgG antibodies [37]. Moreover, the use of a Au gate-equipped nanopore−FET has been demonstrated to allow the synchronised detection of single molecule events in both nanopore and gate channels [38]. With a different approach, nanometric FET sensors were obtained on the tip of spear-shaped dual-carbon nanoelectrodes by electrodeposition of a PPy channel. By means of this nano biosensor, real-time monitoring of extracellular acidity in the microenvironment of cancer cells was carried out and, after binding hexokinase to the PPy channel, extracellular ATP concentrations down to 10 nM were detected [39]. All needle-type transistors realised to date are made of PPy, although PEDOT:PSS is a widely employed organic material to realise transistor operating in aqueous environment, and it is the gold standard for the fabrication of OECT [18], thanks to excellent electrical and electrochemical features together with high chemical stability.

Following this approach, a nanosized OECT with spearhead architecture was realised combining single- and double-barrel CNEs and using PEDOT:PSS as conducting polymer. The fabrication procedure of the nano-sized electrodes was optimised in order to obtain a well-defined geometry for the needle-type gate and individually addressable source-drain contacts. A thin film of PEDOT:PSS was electrochemically deposited on top of them to create nanodisk-shaped gate and channel elements, and the fully assembled spearhead OECT was characterised showing the typical *I*_*d*_ modulation upon gating. The needle-type OECT has single cell-compatible size and a high aspect ratio that allows precise positioning in the desired location by means of a macroscopic handle. In order to give a proof of principle of the potentiality of the implemented device, DA sensing was carried out in a wide concentration range showing picomolar detection limit.

## Experimental

### Chemicals and buffers

Dopamine hydrochloride, L-ascorbic acid, 3,4-ethylenedioxythiophene (EDOT), sodium poly(styrenesulfonate) (NaPSS), hexaammineruthenium(III) chloride, potassium hydroxide and monobasic potassium phosphate were obtained from Sigma Aldrich. Potassium chloride was provided by J.T. Baker. Argon (99.999%), propane (technical grade) and n-butane (99.5%) gases were purchased from Air Liquide. All chemicals were used without any purification. To avoid contaminations, phosphate buffer solution (PBS) and dopamine solutions were prepared using Milli-Q type water (conductivity of 0.055 μS cm^−1^) purified by a water purification system (SG Water, Germany).

### Nanoelectrodes fabrication and characterisation

Nanoelectrodes were fabricated following a previously reported procedure [15] and detailed description is provided in the Supporting Information. Briefly, single- and double-barrel quartz theta capillaries were pulled with a P-2000 laser puller (Sutter Instruments)**,** and a custom-made pyrolysis setup was used to fill the nanopipettes with carbon, thus obtaining single- and double-barrel carbon nanoelectrodes (sbCNEs and dbCNEs). An additional step was necessary to obtain dbCNEs with controlled geometry and size consisting in focused ion beam (FIB) milling. The resulting sbCNEs and dbCNEs were electrochemically characterised by cyclic voltammetry (0.1 < E < − 0.4 V vs Ag/AgCl/3 M KCl; 0.025 V s^−1^) with [Ru(NH_3_)_6_]^3+^ as redox probe. The apparent sizes in nm were approximated from the recorded steady state current in pA. One copper wire was inserted into each barrel from the back to make a connection with the pyroly**s**ed carbon. The reference electrode (RE) was always a Ag/AgCl/3 M KCl electrode. Electrochemical characterisation was performed inside a Faraday cage using a VA-10 voltammetric amplifier (npi electronic) in a two-electrode setup. In this case, the RE also served as counter electrode. The redox mediator solution was prepared using [Ru(NH_3_)_6_]Cl_3_ with a concentration of 5 mM in 0.1 M KCl aqueous solution. The actual size and geometry of the electrodes were assessed by scanning electron microscopy (SEM), which was performed using an environmental scanning electron microscope (eSEM) from FEI Quanta 3D with 20 kV accelerating voltage, equipped with an Energy Dispersive X-ray (EDX) **a**nalyser.

### OECT fabrication with needle-like geometry

Electrodeposition of the polyelectrolyte complex PEDOT:PSS was carried out to coat the carbon nanotips with the electroactive polymer film. Electrochemical deposition was performed inside a Faraday cage using a Jaissle bipotentiostat in a three-electrode setup. A Ag/AgCl/3 M KCl electrode served as reference electrode and a Pt wire acted as counter electrode. The polymerisation solution was prepared by dissolving 10 mM EDOT monomer and 0.1 mM NaPSS as counter ion and supporting electrolyte in deionized water. Before performing the electrodeposition, the solution was kept under magnetic stirring and deaerated under Ar flow for 20 min. When a sbCNE was employed as working electrode, one copper wire was inserted into the barrel to make a connection with the pyrolysed carbon; otherwise, in case of a dbCNE, two copper wires shorted together were inserted into the barrels to make a connection with the pyrolysed carbon and thus apply the potential to both barrels.

Potential pulsing was used for the electrodeposition and a combined protocol, in which a constant potential of 1.2 V vs RE was applied for 200 ms followed by 0 V vs RE for 500 ms, was repeated for two or three times. To assess the formation of the electrodeposited polymer film, the electrodes were imaged and characterised by SEM-EDX. Enhanced capacitive currents in buffer solution and electrocatalytic properties towards DA due to the presence of the polymer layer were verified by CV for the nanogate electrodes. For the nanosized channels, the connection between source and drain was assessed by recording the current flowing through the connecting channel upon application of a constant drain bias.

### Characterisation of needle-type OECTs

**Fig. 1:**
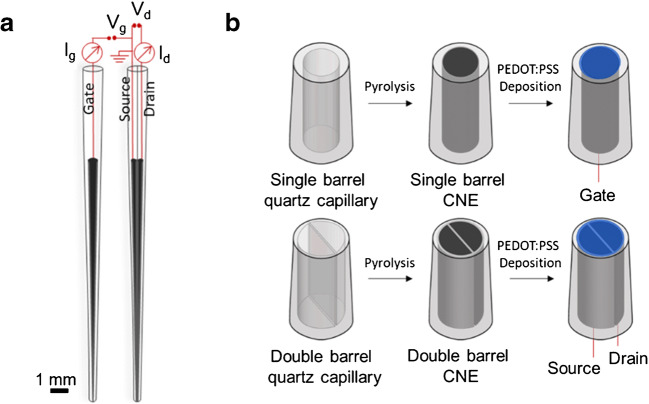
Device schematics. **a** Configuration of the needle-type CNEs. **b** Fabrication steps of PEDOT:PSS spearhead gate and channel

The OECT components were assembled as shown in Fig. 1a and kept at a fixed distance of 0.5 cm during analyses in a Faraday cage. I-V characteristics were recorded in 0.1 M phosphate buffer solution (pH 7.4) using a bipotentiostat to apply gate and drain voltages and to record the corresponding currents. The needle-type gate electrode was connected to the WE1 of the bipotentiostat**;** the drain was connected to WE2 and RE/CE were shorted to the source electrode (grounded).

### Dopamine sensing with needle-type OECTs

The same experimental setup described for the OECT characteri**s**ation was employed to assess the OECT response to DA in an electrochemical cell containing 5 mL buffer solution (0.1 M PBS). A fixed potential was applied to the drain (− 200 or − 300 mV), while either a potentiodynamic wave (0 < *V*_*g*_ < − 600 mV) or a fixed potential (− 900 mV) was applied to the gate electrode for the threshold voltage and the *I*_*d*_/*t* measurements, respectively. To avoid contaminations, acid-cleaned glassware was used. A soft nitrogen flux was used to gently mix the solution during DA additions.

## Results and discussion

Schematics of the needle-type OECT are presented in Fig. 1. The OECT channel is a thin film of PEDOT:PSS bridging the spearhead source and drain carbon nanoelectrodes. A gate voltage (*V*_*g*_) is applied to the spearhead gate electrode, which is coated by the same semiconducting polymer, to modulate the current *I*_*d*_ flowing through the OECT channel due to the applied *V*_*d*_ (Fig. 1a). The fabrication steps involved in the realisation of the OECT components are illustrated in Fig. 1b. Single- and double-barrel carbon nanoelectrodes are obtained after pyrolytic decomposition of a butane and propane gas mixture to yield graphitic carbon deposited inside the pulled quartz capillaries. As for the dbCNE, a nanometre-sized quartz wall separates the two nanoelectrodes that are individually addressable and can be therefore referred to as source and drain terminals.

Afterwards, PEDOT:PSS is electrodeposited on the tip of the spearhead CNEs. PEDOT:PSS was the material of choice because of its biocompatibility [19, 20], mixed conductivity corroborated by reversible electrochemical doping [21, 22], electrocatalytic activity towards redox active molecules, good stability and film-forming properties [40]. In the dbCNE tip, the semiconducting polymer film connecting source and drain forms the OECT channel. A nanodisk-shaped PEDOT:PSS gate electrode is generated from the sbCNE. In the following, fabrication and characterisation procedures optimised for the two needle-type OECT components, i.e. gate and channel, are discussed in detail.

### Needle-type gate electrode

**Fig. 2:**
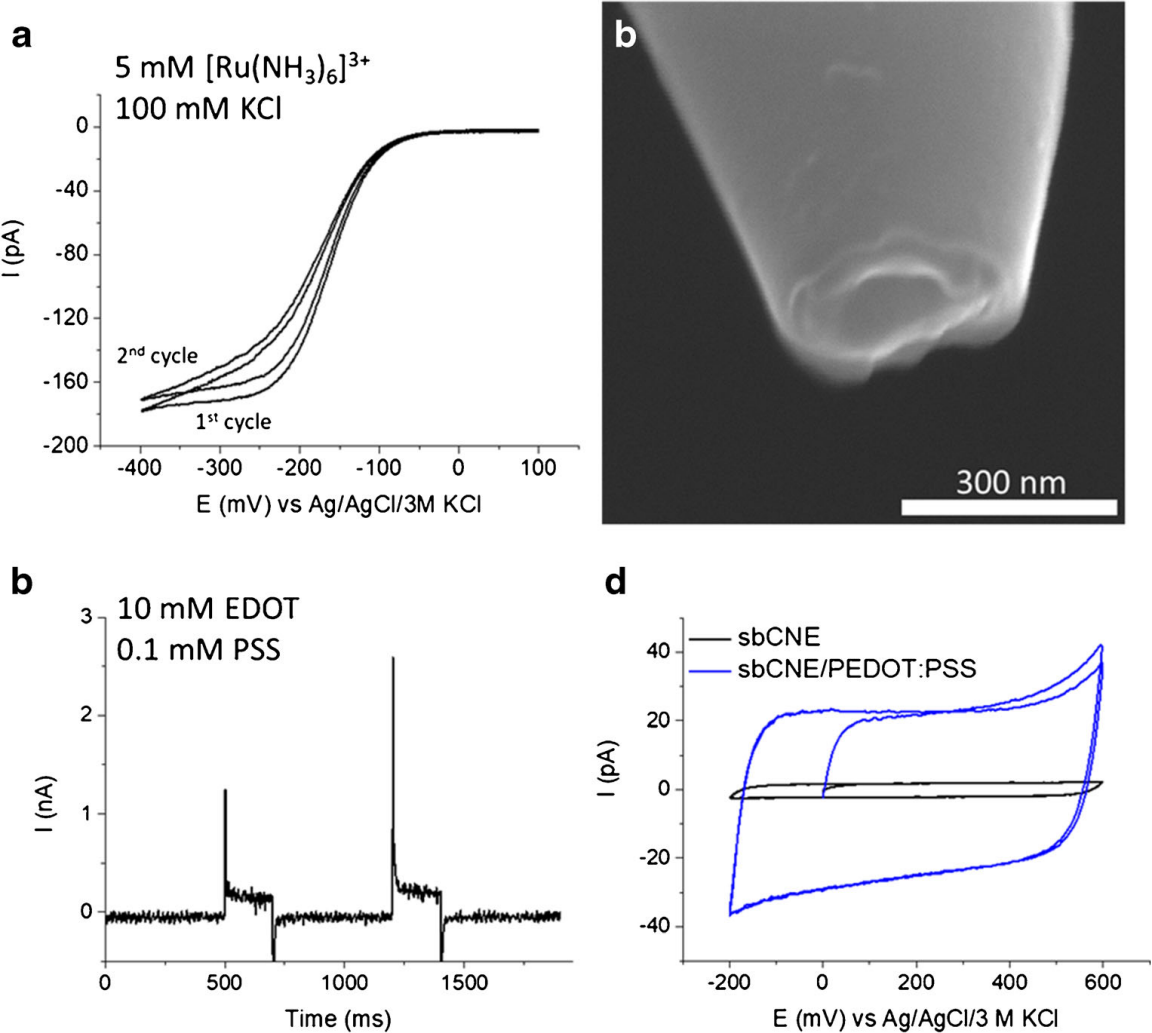
PEDOT:PSS spearhead gate electrode. **a** Electrochemical characterisation of the sbCNE. Scan rate 25 mV s^−1^. **b** SEM picture of the sbCNE. **c** PEDOT:PSS pulse electrodeposition at sbCNE. For each pulse, *E*_app_ = 1.2 V vs Ag/AgCl/3 M KCl for 200 ms. **d** CV characterisation of sbCNE/PEDOT:PSS electrode in phosphate buffer. Scan rate 50 mV s^−1^

After pyrolytic carbon decomposition, the electrochemically active surface of the CNE was evaluated from the steady-state current (*i*_ss_) generated by the reduction of the redox probe [Ru(NH_3_)_6_]^3+^ (Fig. 2a).

However, due to the shape uncertainty at the nanometre scale, estimations made from classical expressions correlating *i*_ss_ and electrode geometry, as well as assumptions on the basis of well-defined sigmoidal voltammograms, are scarcely predictive [15]. For this reason, the actual geometry of nanoscale sbCNEs was always assessed by SEM (Fig. 2b). SEM pictures reveal a hollow quasi-disk structure of the tip, with a radius of 139 ± 3 nm. sbCNEs were coated by a thin film of semiconducting polymer to yield polymeric gate electrodes for the OECT. To this end, cyclic voltammetry (CV) and pulse profiling were compared to carry out PEDOT:PSS electrodeposition, and the effect of the potential waveform as well as monomer concentration was evaluated. Examples of the resulting electrodes are reported in Fig. S2. Upon potential cycling, undesired bunchy structures were obtained, whose size increases at lower scan rates and higher monomer concentration. In contrast, pulse deposition allowed adequate control of the deposition process and led to the formation of thin films on top of the CNEs tip. Therefore, two potential pulses of 200 ms (*E*_app_ = 1.2 V vs Ag/AgCl/3 M KCl) were applied to the sbCNE in the polymerisation solution containing EDOT (10 mM) and the counterion PSS (0.1 mM) (Fig. 2c). A substantial increase of the capacitive current recorded before and after PEDOT:PSS deposition confirmed the growth of the polymer film on the sbCNE (Fig. 2d). A roughly tenfold increase of the double-layer charge is found as compared with the bare sbCNE.

### Needle-type channel

**Fig. 3:**
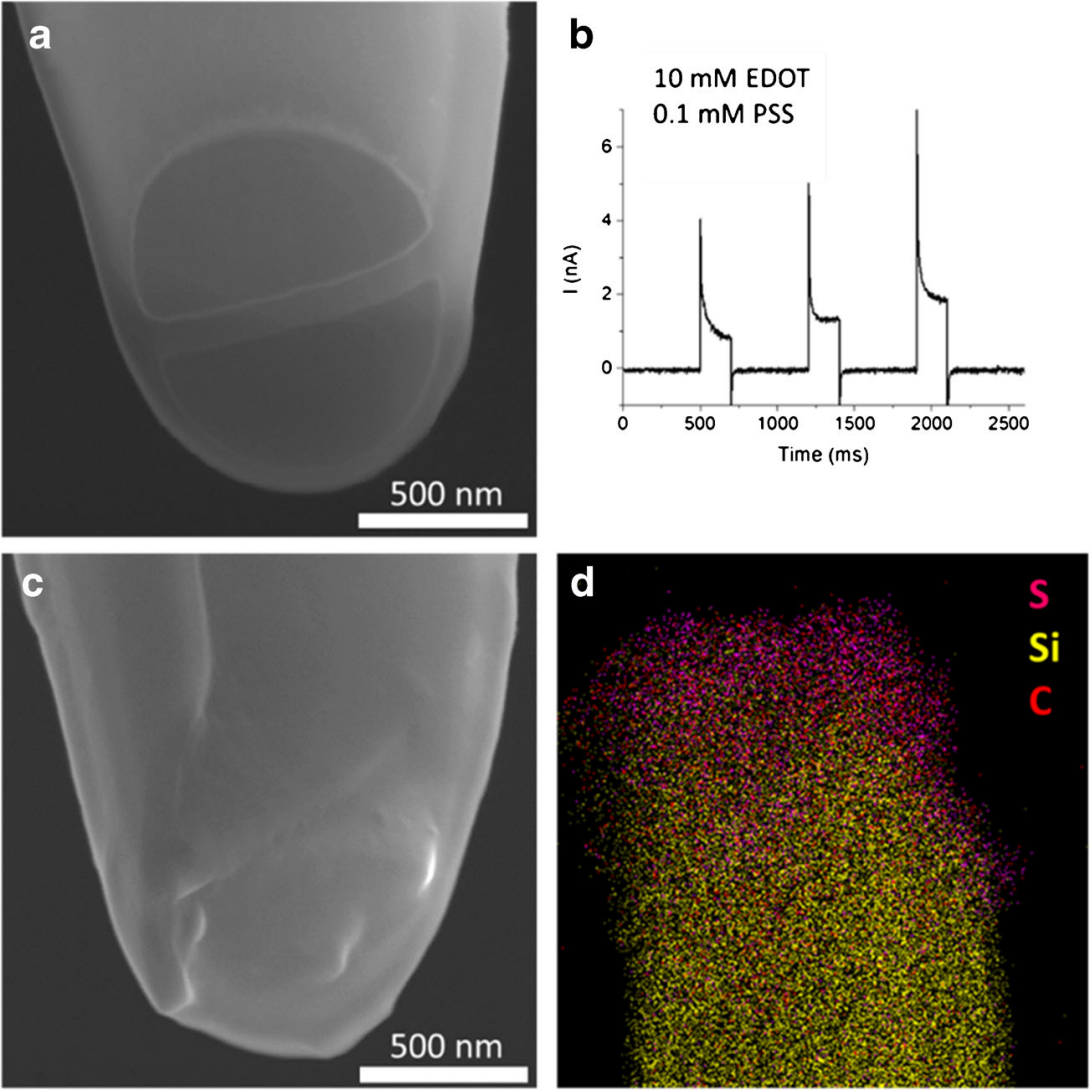
PEDOT:PSS spearhead channel. **a** SEM picture of the dbCNE. **b** I vs time profile recorded during PEDOT:PSS pulse electrodeposition on dbCNE. **c** SEM picture and **d** EDX map of the PEDOT:PSS channel bridging the two barrels

The optimised procedure for dbCNEs fabrication includes the use of focused ion beam milling to yield electrodes with controlled geometry and size (Fig. 3a). Indeed, the electrodes obtained right after pyrolysis present more defects and poor size reproducibility with respect to sbCNEs (see Figs. S4 and S5). By introduction of the additional FIB milling step, it is possible to cut off the dbCNE tips with carbon overgrowth (Fig. S5A), cracks (Fig. S5B) and recessed carbon edges (Fig. S5C), leading to regular and well-defined shapes. The resulting two barrels are individually addressable and separated by a thin quartz wall of 48 ± 8 nm. They can be virtually seen as source and drain electrodes. Based on the considerations made for the sbCNEs, a pulse profile (Fig. 3b) was chosen to deposit a film of PEDOT:PSS that bridges the two barrels to form the semiconducting channel. In this case, at least three pulses are typically needed to obtain a stable polymer film that provides the desired electrical connection between source and drain. Figure 3a and c show SEM images of a dbCNE before and after PEDOT:PSS electrodeposition, respectively, where the deposited layer covers completely the quartz wall and establishes a nanojunction between the barrels. The presence of the polymer channel is further assessed by SEM-EDX mapping (Fig. 3d), where sulfur, arising from the tiophene rings of PEDOT and sulfonate groups of PSS, is also found on the quartz wall of the dbCNE after electrodeposition.

### Fabrication of the needle-type OECT

**Fig. 4:**
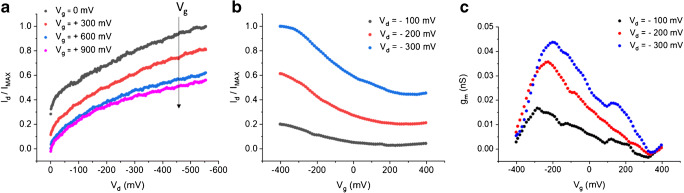
OECT characterisation in buffer solution. **a** Output characteristics recorded at 20 mV s^−1^. **b** Transfer curves recorded at 100 mV s^−1^ and **c** correspondent transconductance vs *V*_*g*_ plot. All measurements were performed in 100 mM PBS

Following the abovementioned fabrication procedures, all-PEDOT:PSS spearhead gate electrodes and channels with a diameter of about 300 and 900 nm, respectively, were obtained. Combination of the two electronic components leads to the fully functioning, needle-type OECT configuration illustrated in Fig. 1a. The transistor was characterised by recording I-V curves in buffer solution (Fig. 4). Output characteristics in Fig. 4a show the *I*_*d*_ vs *V*_*d*_ curves recorded at different voltages applied to the gate electrode. *I*_*g*_ is an ionic current that travels across the electrolyte and affects the electronic current that flows through the channel, depending on the applied *V*_*g*_. Due to a positive gate bias, PEDOT oxidation takes place at the gate, and the electrons extracted here are injected into the channel leading to the reduction of PEDOT^+^. To maintain electroneutrality, cations from the electrolyte are injected into the polymer channel and compensate the negatively charged sulfonate groups of PSS. This process is reversible and is described as electrochemical doping (Reaction 1):

1 $$ {\mathrm{PEDOT}}^{+}:{\mathrm{PSS}}^{-}+{e}^{-}+{M}^{+}\leftrightarrows \mathrm{PEDOT}+{M}^{+}{\mathrm{PSS}}^{-} $$

Since the oxidised centres of the polymer are the charge carriers, reduction of PEDOT^+^ corresponds to holes extraction from the OECT channel, thus leading to a decreased conductivity ultimately causing the observed *I*_*d*_ modulation. On the contrary, a negative gate bias would cause PEDOT^+^ reduction at the gate electrode, while electrochemical oxidation takes place in the channel (holes injection) leading to an increase of *I*_*d*_. [41, 42] Despite the sub-micrometric size, so-called “short-channel effects” [35] resulting, for example, in a lack of current saturation or non-linear output characteristics, are not observed. OECT operation is well described by the transfer characteristics in Fig. 4b, where the output *I*_*d*_ is measured while linearly scanning the gate voltage, at fixed *V*_*d*_. The OECT amplification is expressed by the transconductance *g*_*m*_, which is the effective parameter to quantitatively describe the modulation of the channel current upon gating (Fig. 4c). It has to be noted that *g*_*m*_ values are significantly lower than those reported for other nanometric OECTs, which are typically in the mS range [34, 35]. However, given the round-shaped channel geometry and the fact that the polymer films were obtained by electrodeposition, in contrast to typically planar, rectangular channels deposited from commercial PEDOT:PSS inks, in comparison with the state-of-the art, might not be meaningful. Repeatability studies of the OECT response upon reversible current modulation, using either a needle-type PEDOT:PSS gate electrode or a Ag/AgCl microelectrode, in buffer and in the presence of ascorbic acid (AA), are reported in Fig. S6.

While more efficient modulation of the *I*_*d*_ is obtained with the non-polarisable gate microelectrode, a reversible OECT behaviour in the output characteristics is seen only when a polymeric nanogate is used (Fig. S6A, B). AA was exploited in these preliminary experiments because it is a benchmark in the response evaluation of electrochemical devices based on polythiophenes.

### Dopamine sensing with the needle-type OECT

**Fig. 5:**
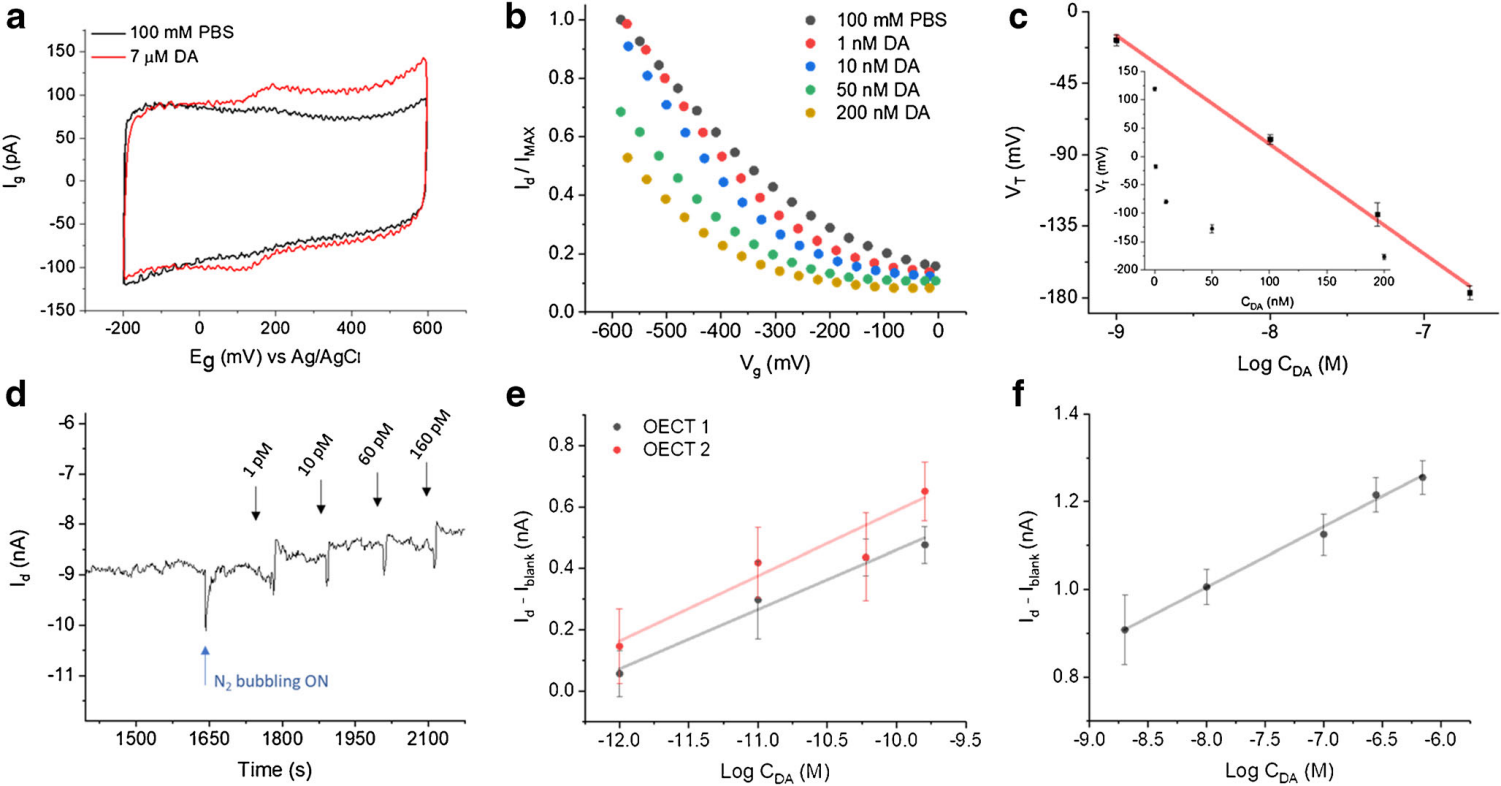
Dopamine detection with the needle-type OECT. **a** Electrochemical response of the PEDOT:PSS gate electrode to DA. Scan rate 100 mV s^−1^. **b** Transfer curves recorded in presence of increasing amounts of DA. *V*_*d*_ = − 200 mV, scan rate 10 mV s^−1^. **c** Threshold voltage response to the concentration (inset) and logarithm of DA concentration in the nM range. Standard deviations are given as error bars. **d***I*_*d*_ vs time curve recorded during increasing additions of DA in the range from 1 to 160 pM (*V*_*g*_ = − 900 mV; *V*_*d*_ = − 300 mV). Calibration curves obtained **e** from 1 to 160 pM and **f** from 2 to 700 nM DA

Figure 5a shows the voltammogram obtained at a spearhead PEDOT:PSS gate electrode in buffer and after addition of DA. A couple of well defined and reversible peaks, with *E*p_A_ = + 194 mV and *E*p_C_ = + 123 mV vs Ag/AgCl/3 M KCl appears due to the reversible redox reaction of DA at the PEDOT:PSS electrode. As long as a chemical species is able to take part in the doping/de-doping mechanism that rules the transistor operation, it can be detected by the OECT. In the case of a redox active molecule like DA, its oxidation can be favoured either at the polymer gate or channel, depending on their relative electrochemical potentials. Indeed, *V*_*g*_ determines the electrochemical potentials of gate (*E*_*g*_) and channel (*E*_ch_), thus establishing at which OECT element DA oxidation to dopamine quinone (DQ) takes place. When a positive *V*_*g*_ is applied, *E*_*g*_ is higher than the electrochemical potential of the source (*E*_*s*_) and DA oxidation is favoured at the gate. Conversely, for negative *V*_*g*_ values, the reaction mainly takes place at the channel because *E*_*g*_ is lower than *E*_*d*_ [28, 42]. This phenomenon can be exploited for sensing and, with the aim to show a potential applicability of the needle-type OECTs, the proof-of-concept detection of DA was explored either by using a potentiodynamic gate bias or applying fixed *V*_*d*_ and *V*_*g*_. Transfer characteristics were recorded in the presence of increasing DA concentrations in the nM range upon application of a linear ramp to the gate electrode (Fig. 5b). If *V*_*g*_ is sufficiently negative, DA oxidation is favoured at the OECT channel, directly provoking the depletion of carriers with the expected *I*_*d*_ variation, according to Reaction 2

2 $$ 2{\mathrm{PEDOT}}^{+}+\mathrm{DA}\to 2\mathrm{PEDOT}+\mathrm{DQ}+2{H}^{+} $$

The presence of an oxidisable species like DA in solution can be imagined as an “extra gate” switching off the OECT channel. Consequently, the higher the concentration of DA, the more difficult it is to invoke a current flow through the channel and, consequently, the transfer curves shift downwards with increasing DA concentration. The gate bias that is needed to turn the transistor on is known as threshold voltage, *V*_*T*_, and it can be extracted from the linear region of the transfer curves as the *x*-axis intercept [43, 44]. As reported in Fig. 5c, *V*_*T*_ scales linearly with the logarithm of DA concentration with a sensitivity of (69 ± 2) mV decade^−1^ (*R*^2^ = 0.996). Sensing performances of the needle-type OECTs were also tested upon application of fixed *V*_*g*_ and *V*_*d*_ (− 900 and − 300 mV, respectively) by adding increasing amounts of DA to the buffer solution under controlled conditions, while *I*_*d*_ was measured over time (Fig. 5d). Analysis of the signal stabilisation before DA additions is given in Fig. S7. As expected, each addition was followed by an *I*_*d*_ decrement due to the depletion of holes from the channel boosted by DA oxidation (Fig. 5d). The modulated current exhibits a logarithmic dependence on the analyte concentration. In Fig. 5e, we report the performance of two sensors in the low pM range, where the sensitivities are (190 ± 20) pA decade^−1^ (OECT 1, *R*^2^ 0.984) and (210 ± 40) pA decade^−1^ (OECT 2, *R*^2^ 0.932). The detection limit (LoD) calculated by the 3*σ*/*m* criterion (where *σ* is the standard deviation of the blank and *m* is the slope of the calibration curve in the pM range) was 1 pM. A second linear range was found at higher DA concentrations, i.e. from 2 to 700 nM, where the needle-type OECT shows a sensitivity of (138 ± 6 pA) decade^−1^ (*R*^2^ 0.994) (Fig. 5f). While the reproducibility among different sensors is limited to the uniqueness of each needle-type device, the initial performance is not recovered after real-time measurement in the whole concentration range under investigation, thus suggesting a disposable use of these tools.

Table 1 reports the figures of merit of the state-of-the-art nanometric electrochemical sensors allowing spatially resolved DA detection. It is worth noting that the transistor architecture provides the needle-type OECT with superior sensitivity, highlighted by the especially low detection limit, with consequent simplification of both experimental setup and readout electronics. However, the uniqueness of each spearhead sensor and the unaddressed selectivity issue place the here reported devices at a primal stage of development with respect to other literature reports

**Table 1:** Summary of nanometric electrochemical sensors for DA reported in literature

Sensor type	Sensor size	Sensing material	Detection method	LoD (nM)	Linear range (M)	Selectivity (tested species)	Ref.
IDEs array (chip)	150 nm width, 300 nm pitch	Au	RC	620	10^−7^–10^−4^	Yes (AA)	[45]
Au NE (needle-type)	50 nm radius (Au disk w/o glass sheath)	Au clusters	CA	5.2	(0.01–2.55) × 10^−6^	Yes, Nafion coated (UA, AA, NE, E, DOPAC)	[46]
Au NE (needle-type)	200 nm (tip radius)	PANi	DPV	100	(0.3–200) × 10^−6^	Yes (AA, Na^+^ K^+^, Mg^2+^, Ca^2+^, G, Lys, GA, C))	[47]
Au NE (chip)	30–500 nm width	Au	LSV	128	(0.004–1.012) × 10^−3^	Yes (AA)	[48]
CFNE (needle-type)	100–300 nm (fibre diameter)	Carbon	DPV	40	N/A	No	[49]
CNPE (needle-type)	250 nm (tip diameter)	Carbon	FSCV	25	(0.1–10) × 10^−6^	No	[14]
CNE-based OECT (needle-type)	300 nm, 900 nm (gate channel diameter)	PEDOT:PSS	OECT - potentiostic	0.001	(1–160) × 10^−12^ (2–700) × 10^−9^	No	This work

*IDEs*, intedigitated electrodes; *NE*, nanoelectrode; *CFNE*, carbon fiber nanoelectrode; *CNPE*, carbon nanopipette electrode; *CNE*, carbon nano electrode; *OECT*, organic electrochemical transistor; *RC*, redox cycling; *CA*, chronoamperometry; *DPV*, differential pulse voltammetry; *LSV*, linear sweep voltammetry; *FSCV*, fast scan cyclic voltammetry; *AA*, ascorbic acid; *UA*, uric acid; *NE*, norepinephrine; *E*, epinephrine; *DOPAC*, 3,4-dihydroxyphenylacetic acid; *G*, glucose; *L*, lys; *GA*, glutamic acid; *C*, citric acid

Indeed, one of the major analytical challenges in DA detection is the ubiquitary presence of interfering species in brain tissues, such as AA [50], whose electrochemical oxidation at a PEDOT:PSS electrode occurs in a potential window very close to DA (Fig. S8). Concurrently, the sluggish kinetics associated to AA oxidation can be exploited to suppress its faradaic contribution at high scan rates. This approach has been successfully employed for the selective detection of DA with a macroscopic all-PEDOT:PSS OECT using a potentiodynamic gate bias [28] and could be applied to the needle-type OECT as well.

## Conclusions

In this work, the first all-PEDOT:PSS OECT with needle-type architecture was realised using single- and double-barrel carbon nanoelectrodes generating a nanometre-sized electrochemical device that combines superior sensing performances with high spatial resolution. Not only the typical OECT geometry was significantly downscaled, reaching the sub-micrometre range, but also the conventional chip-like architecture was revolutionised to fabricate a spearhead tool having cell-compatible dimensions that could be positioned in the desired location using a macroscopic handle. The fully assembled, needle-type OECT showed reversible current modulation upon gating across the electrolyte medium, with good repeatability and stability of the transistor response. Further studies are needed to correlate the transistor behaviour and figures of merit with the features of consolidated chip-like organic devices. In order to demonstrate the potentiality of the needle-type OECT, its sensing performance was tested in the proof-of-concept detection of Dopamine in buffer solution. On one hand, the transistor architecture provides the needle-type OECT with superior sensitivity and considerably simplifies both experimental setup and readout electronics with respect to conventional electroanalytical methods. Additionally, if compared to a planar OECT counterpart, the spearhead-type geometry which we propose here may be suitable in the future for being stereotaxically inserted into deeper brain regions without causing tissue damage and reducing neuroinflammation due to its shape and the very small overall dimensions. On the other hand, the uniqueness of each spearhead sensor and the unaddressed selectivity issue place these devices at a primal stage of development with respect to other literature reports. Several strategies could be used to improve the needle-type OECT in view of challenging bioelectronic applications, such as locally resolved single-cell analyses, detection of ion fluxes across the cell membranes and description of biomolecules distribution and dynamics in real-time. For instance, the ease of functionalisation of the organic constituents makes the needle-type OECT a versatile platform for material research, while the use of a potentiodynamic approach during the OECT operation as a sensor could allow selective detection of redox active molecules in biological media.

## Electronic supplementary material

ESM 1 (DOCX 1639 kb).
