# Supplementary material for: Needle-type organic electrochemical transistor for spatially resolved detection of dopamine
Source: Mikrochim Acta. 2020 Jun 9;187(7):378. doi: 10.1007/s00604-020-04352-1 (PMC7283208; doi:10.1007/s00604-020-04352-1)
Supplement: Supplementary file 1 — (DOCX 1639 kb). [file 604_2020_4352_MOESM1_ESM.docx]

**Electronic Supplementary Material**

**Needle-type organic electrochemical transistor for spatially resolved detection of dopamine**

Federica Mariani ^1^, Thomas Quast ^2^, Corina Andronescu ^3^, Isacco Gualandi ^1^, Beatrice Fraboni ^4^, Domenica Tonelli ^1^, Erika Scavetta ^1,*^, Wolfgang Schuhmann ^2,^*

^1^ Dipartimento di Chimica Industriale “Toso Montanari”, Università di Bologna, Viale del Risorgimento 4, 40136 Bologna, Italy.

^2^ Analytical Chemistry - Center for Electrochemical Sciences (CES), Faculty of Chemistry and Biochemistry, Ruhr University Bochum, Universitätsstraße 150, 44780 Bochum, Germany.

^3^ Chemical Technology III, Faculty of Chemistry and Center for Nanointegration (CENIDE); University Duisburg Essen, Carl-Benz-Str. 201, D-47057, Duisburg, Germany.

^4^ Dipartimento di Fisica e Astronomia, Università di Bologna, Viale Berti Pichat 6/2, 40127 Bologna, Italy.

* Corresponding authors. erika.scavetta2@unibo.it; wolfgang.schuhmann@rub.de

Nanoelectrodes fabrication. For the fabrication of nanopipettes, single- and double-barrel quartz theta capillaries (outer diameter 1.2 mm, internal diameter 0.9 mm, Sutter Instruments) were pulled with a P-2000 laser puller (Sutter Instruments) using the following parameters:

Table S1. Pulling parameters optimized for double- and single-barrel nanopipettes fabrication.

| Type | Heat | Filament | Velocity | Delay | Pull |
| --- | --- | --- | --- | --- | --- |
| **Single Barrel** | 800 | 4 | 45 | 130 | 90 |
| **Double Barrel** | 800 | 4 | 45 | 130 | 100 |

as schematically illustrated in Figure S1 A. Afterwards, a custom-made pyrolysis setup [15] was employed to fill the nanopipettes with carbon, thus yielding single- and double-barrel carbon nano­electrodes (sbCNEs and dbCNEs). During pyrolysis (Figure S1 B), the Ar counter-flow was set at a rate of 50 mL min^-1^ with the Ar pressure fixed at 0.5 bar. Pyrolysis gas was introduced as a mixture of propane with a pressure of 3 bar and n-butane with a pressure of 1.1 bar. Coil movement and heating current were simultaneously controlled by a specifically designed control software. Opti­mized temperature profiles used during pyrolysis are shown in Figure S1 C for sbCNEs and dbCNEs. After pyrolysis, the electrode was allowed to cool down under Ar stream for at least additional 35 s to prevent oxidation of carbon at atmospheric conditions.


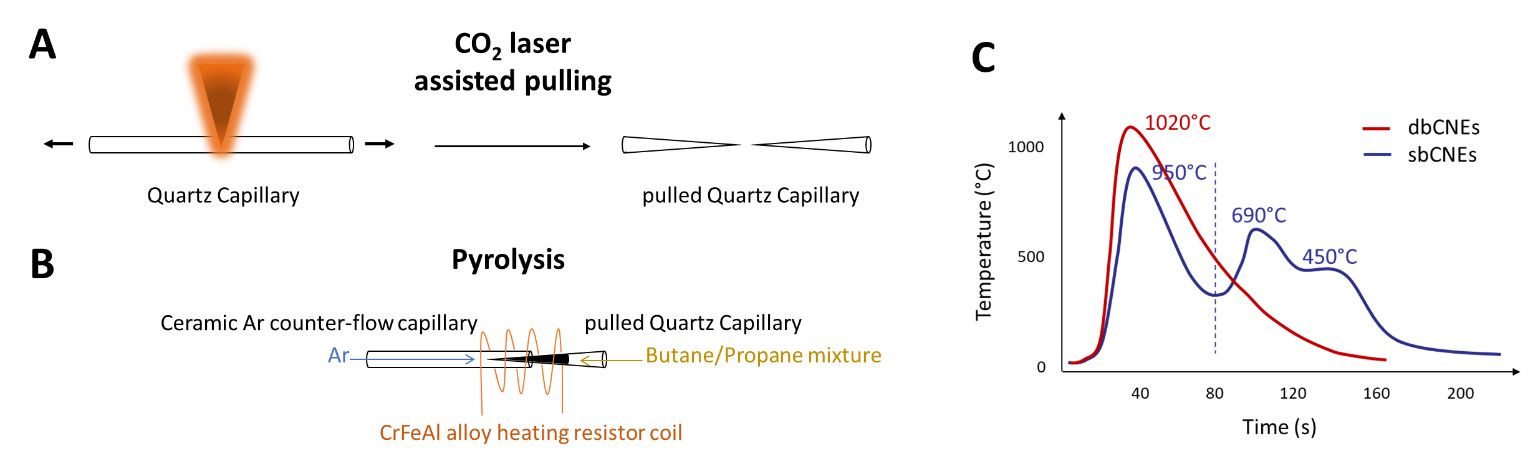


Figure S1. Fabrication of single- and double-barrel CNEs. Schemes of (A) the laser pulling and (B) the pyrolysis steps. (C) Optimized temperature profiles used during pyrolysis.

**Electrochemical deposition of PEDOT:PSS.** Cyclic voltammetry (CV) and pulse profiling were compared for PEDOT:PSS electrodeposition at the CNEs. Together with the potential wave­form, monomer concentration (10 or 5 mM) and other parameters specific to the technique of choice (scan rate and number of cycles for CV; number and duration of pulses for pulse deposition) were varied, while keeping the PSS concentration (0.1 mM), pulse potential (E_app_ = 1.2 V *vs* Ag/AgCl/3M KCl) and CV potential window (0 < E_app_ < 1.2 V *vs* Ag/AgCl/3M KCl) fixed. The pulse potential as well as the anodic potential limit in CV were chosen to trigger the monomer oxidation and promote the polymerization avoiding PEDOT overoxidation. Examples of the electrode’s shape obtained by the two methods are shown in **Figure S2**.


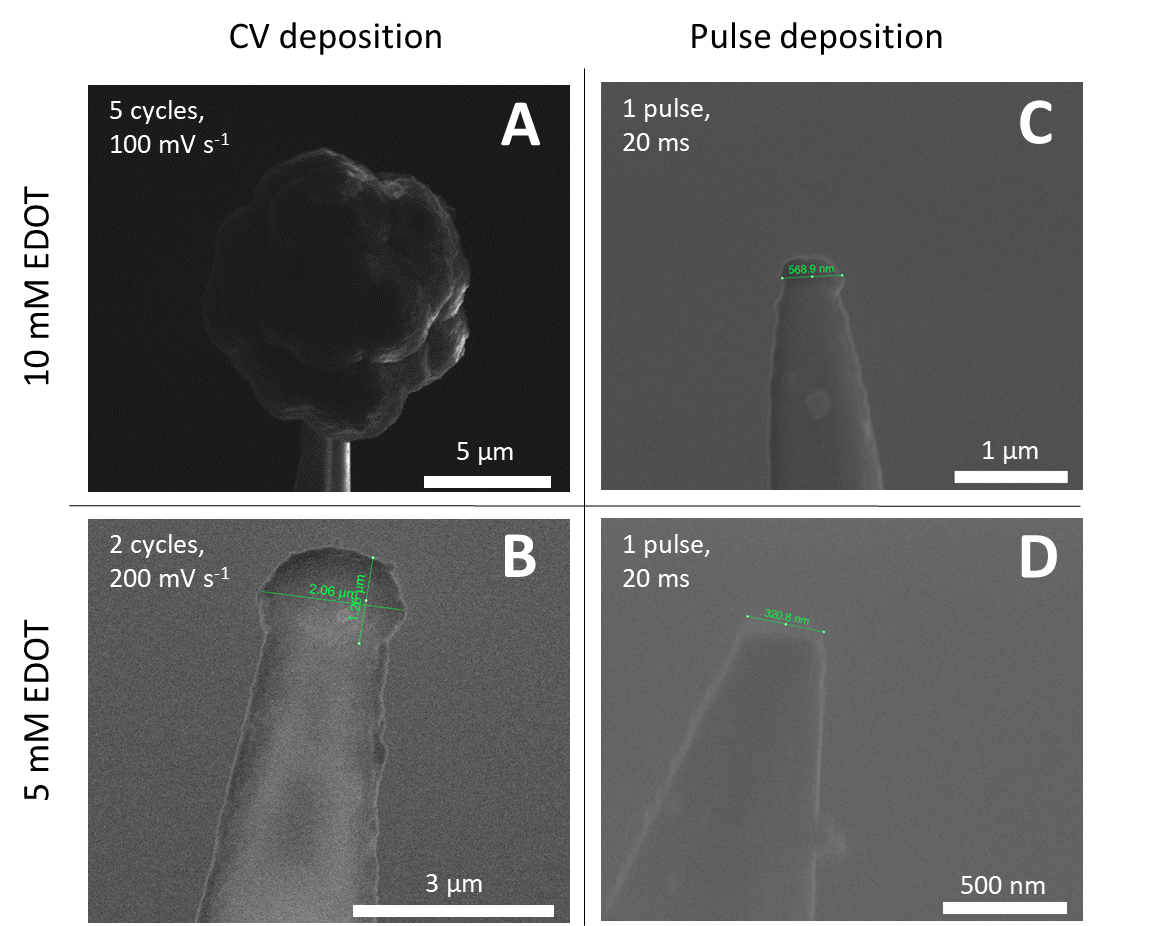


**Figure S2. SEM images of CNEs after PEDOT:PSS electrodeposition.** The depositions were carried out in aqueous EDOT (5 or 10 mM) and PSS (0.1 mM) solutions by means of of either potential cycles in the interval 0 < E_app_ < 1.2 V vs Ag/AgCl/3M KCl (**A**, **B**) or potential pulses with E_app_ = 1.2 V vs Ag/AgCl/3 M KCl (**C**, **D**). Diameters of the PEDOT:PSS deposits estimated by SEM are 10.3 μm (A), 2.1 μm (B), 568.9 nm (C) and 320.8 nm (D).

The shape of the electrode obtained after 2 cycles suggests that the formation of one PEDOT-bubble roughly follows one potential cycle and faster scan rates should produce more discrete deposits (Fig, S2 A, B). The cyclic voltammogram recorded during PEDOT:PSS deposition of the CNE shown in Figure S2 B is reported in Figure S3. It is well-known that EDOT oxidation occurs at higher potentials with respect to PEDOT and the polymer growth is facilitated with each consecutive cycle. The struc­tures obtained after pulse depostion also show globular features when the pulse duration exceeds 300 ms (not shown). However, the time scale in which the electrodeposition occurs (tens/hundreds of milliseconds) is far smaller than the one during CV. This prevents the formation of large aggregates and, upon selection of suitable parameters for pulse duration and frequency it is possible to obtain film-like and controlled depositions (Fig. S2 C, D). Overall, it was noted that using lower monomer concentrations (5 mM) during both CV and pulse deposition, as well as frequent pulses with a shorter duration (20 ms) lead to less stable PEDOT:PSS deposits on the CNEs that often detach during the following measurements. Consequently, the parameter of choice to perform electrodeposition were two and three potential pulses of 200 ms each (E_app_ = 1.2 V vs Ag/AgCl 3M KCl) for sbCNEs and dbCNEs, respectively in the polymerization solution containing EDOT (10 mM) and the counterion PSS (0.1 mM).

**
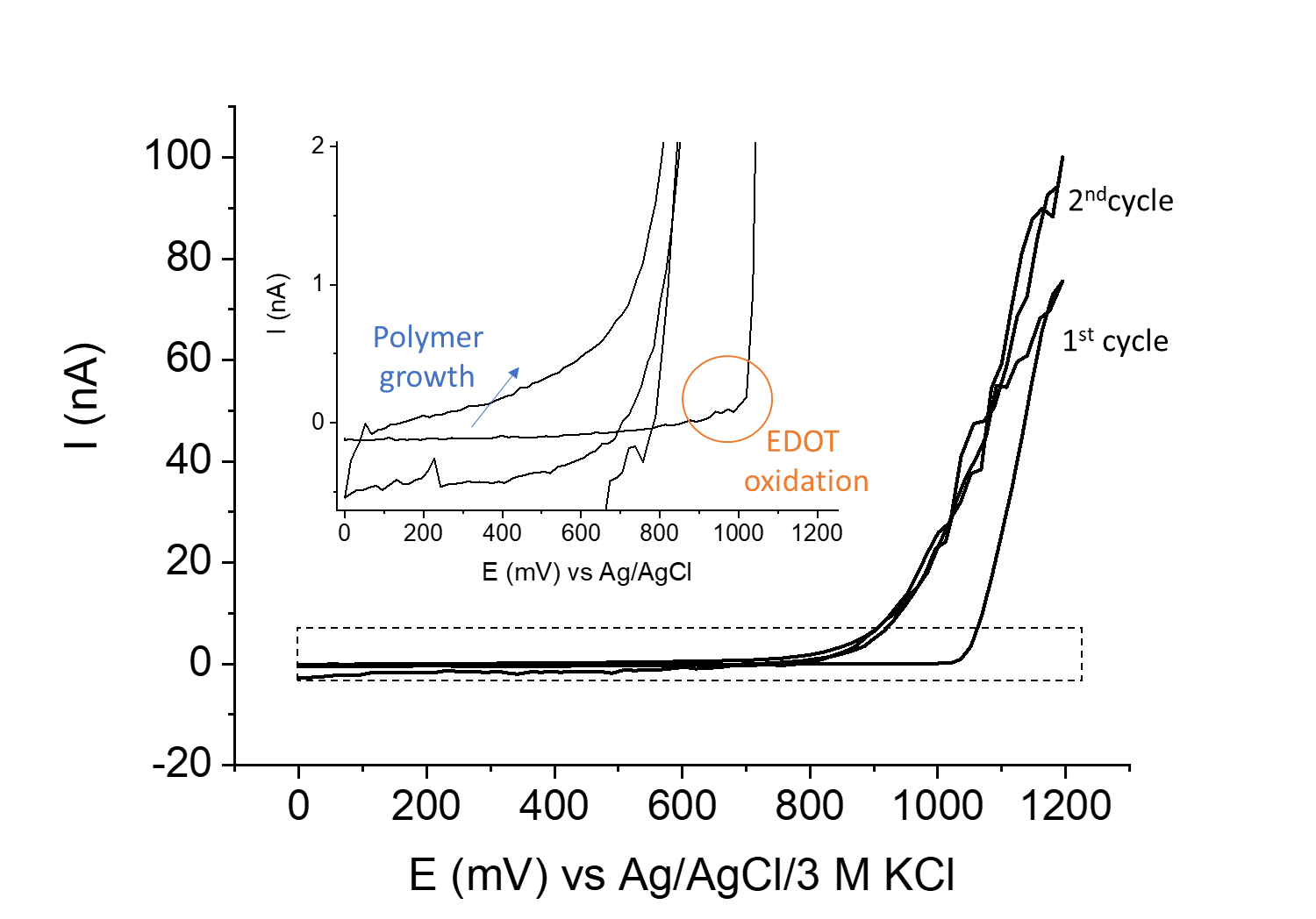
**

**Figure S3. PEDOT:PSS deposition at CNE by CV** to obtain the electrode shown in Figure S4 B. Inset: zoom of the voltammogram region highlighted by the dotted rectangle that shows monomer oxidation and polymer growth upon cycling.

**
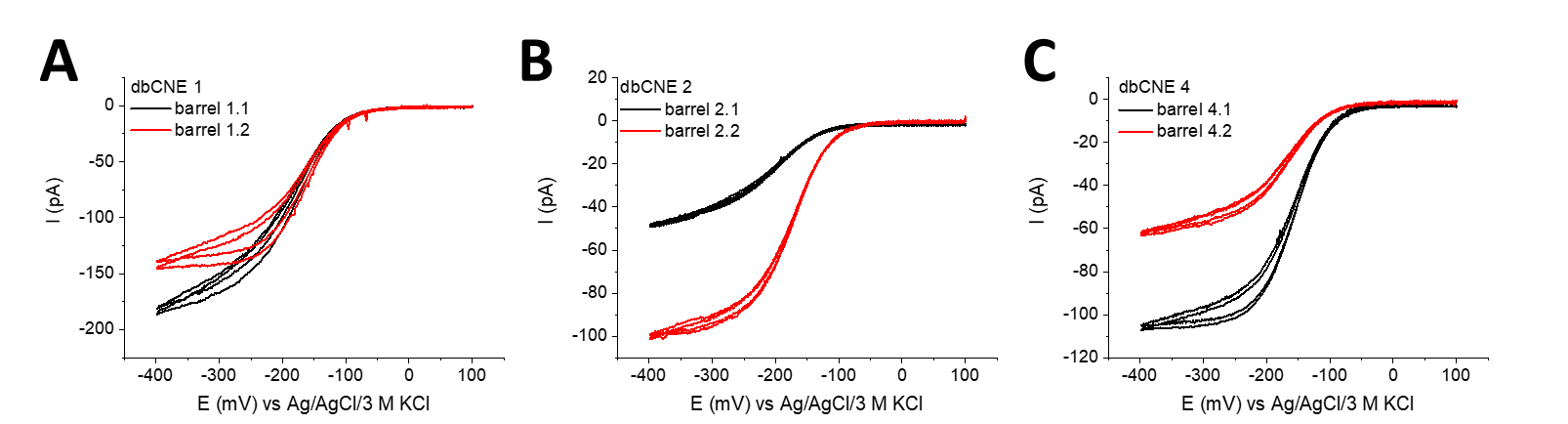
**

**Figure S4. Cyclic voltammograms** recorded at 25 mV s^-1^ in a solution containing 5 mM [Ru(NH_3_)_6_]^3+^ and 100 mM KCl of the dbCNEs right after pyrolysis.


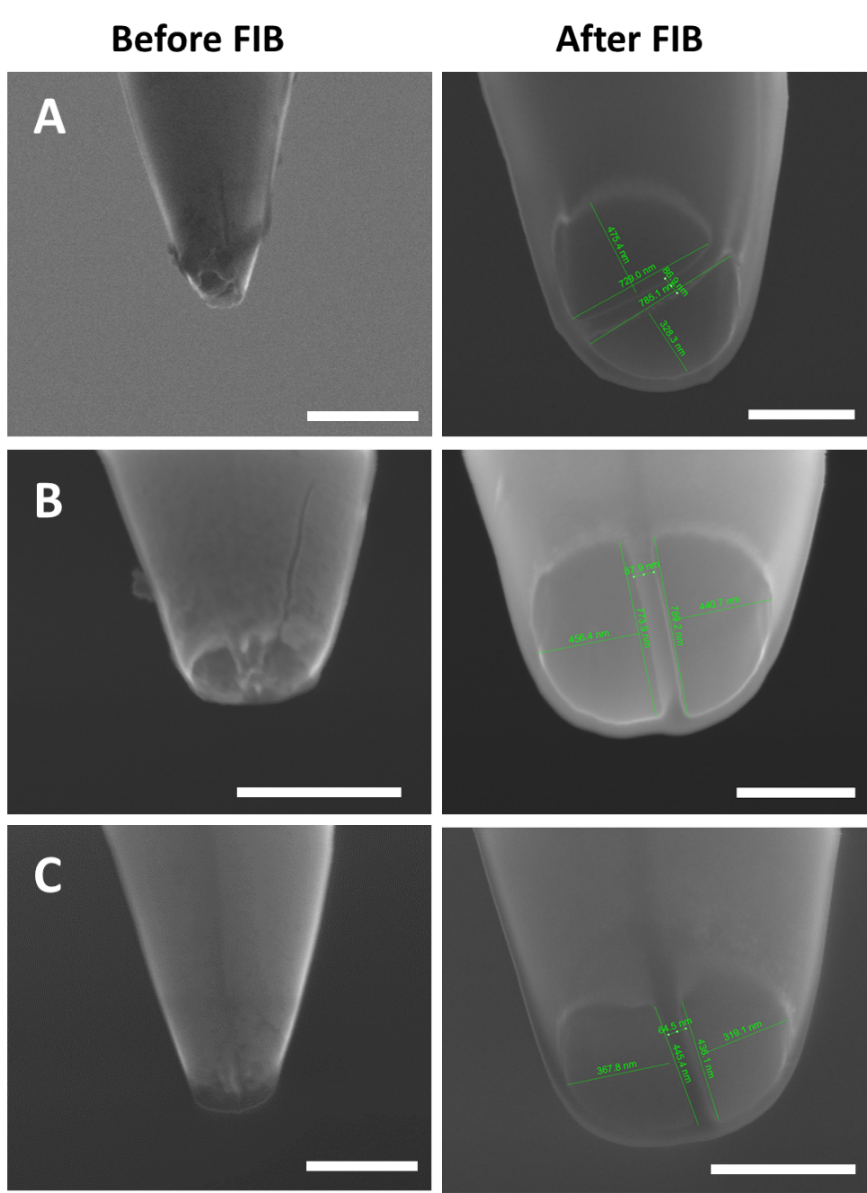


**Figure S5. dbCNEs before and after focused ion beam milling**. SEM pictures of the dbCNEs with carbon overgrowth (A), cracks (B) and recessed carbon edges (C), before and after the FIB milling step. Scale bar: 500 nm.

**
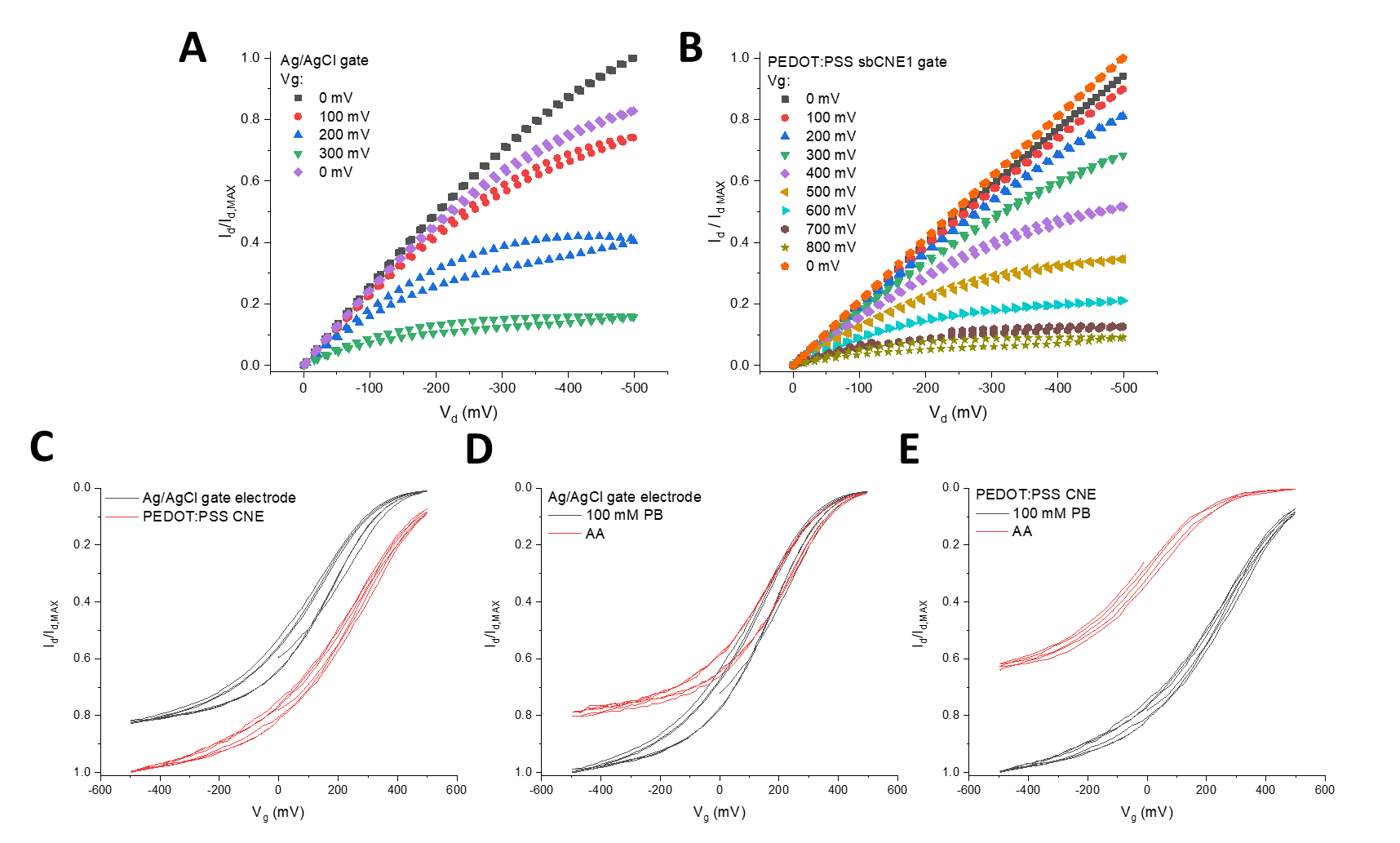
**

**Figure S6. Repeatability of the nano OECT response.** Output characteristics of a nano-sized channel with a Ag/AgCl microelectrode (**A**) or a PEDOT:PSS CNE (**B**) as gate electrode in buffer solution.Transfer characteristics of a nano-sized channel with a Ag/AgCl microelectrode or a PEDOT:PSS CNE as gate electrode in buffer solution and in the presence of 1 mM ascorbic acid (AA) (**C**, **D**, **E**).


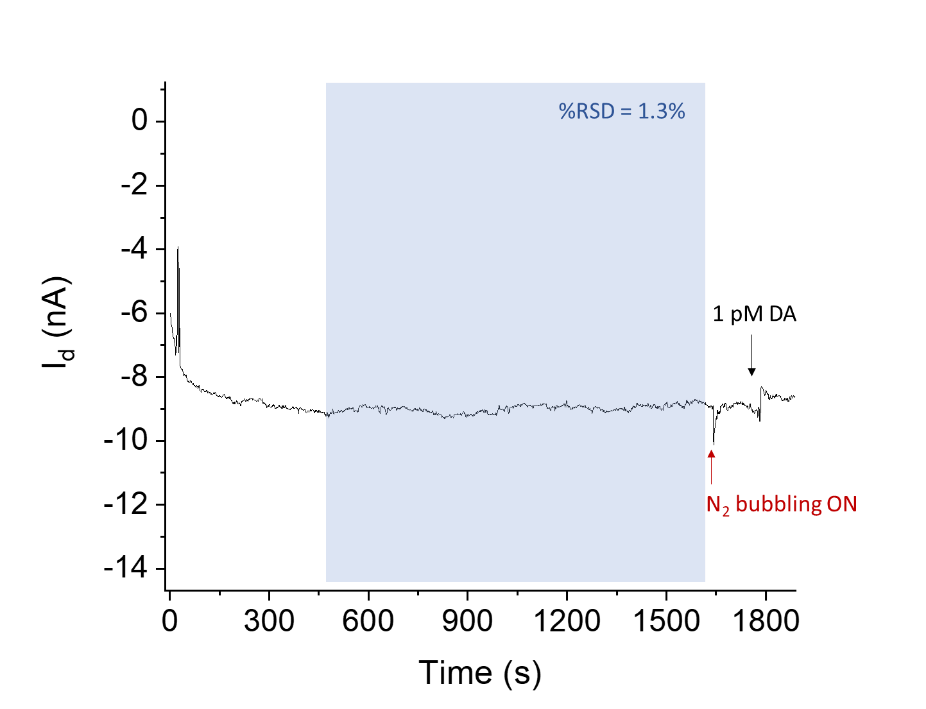


**Figure S7. Stabilization of Id.** I_d_ vs time curve showing the stabilization of the OECT output current upon application of V_g_ = -0.9 V and V_d_ = -0.3 V before DA additions. Current drift over the stabilization window is given as %RSD and is equal to 1.3%.

**Response to Ascorbic Acid.** The response of a PEDOT:PSS gate electrode in binary mixtures containing equimolar DA and AA was studied by CV (Figure S8). The voltammograms recorded at different scan rates (Figure S8 A and B) suggest the sluggish kinetics associated to AA electro-oxidation, whose contribution is less resolved with increased scan rate. In contrast, DA contribution is clearly observed and, despite the presence of AA and the scan rate of 50 mV s^-1^, the electrode response to increasing DA concentrations in solution is linear (Figure S8 C).


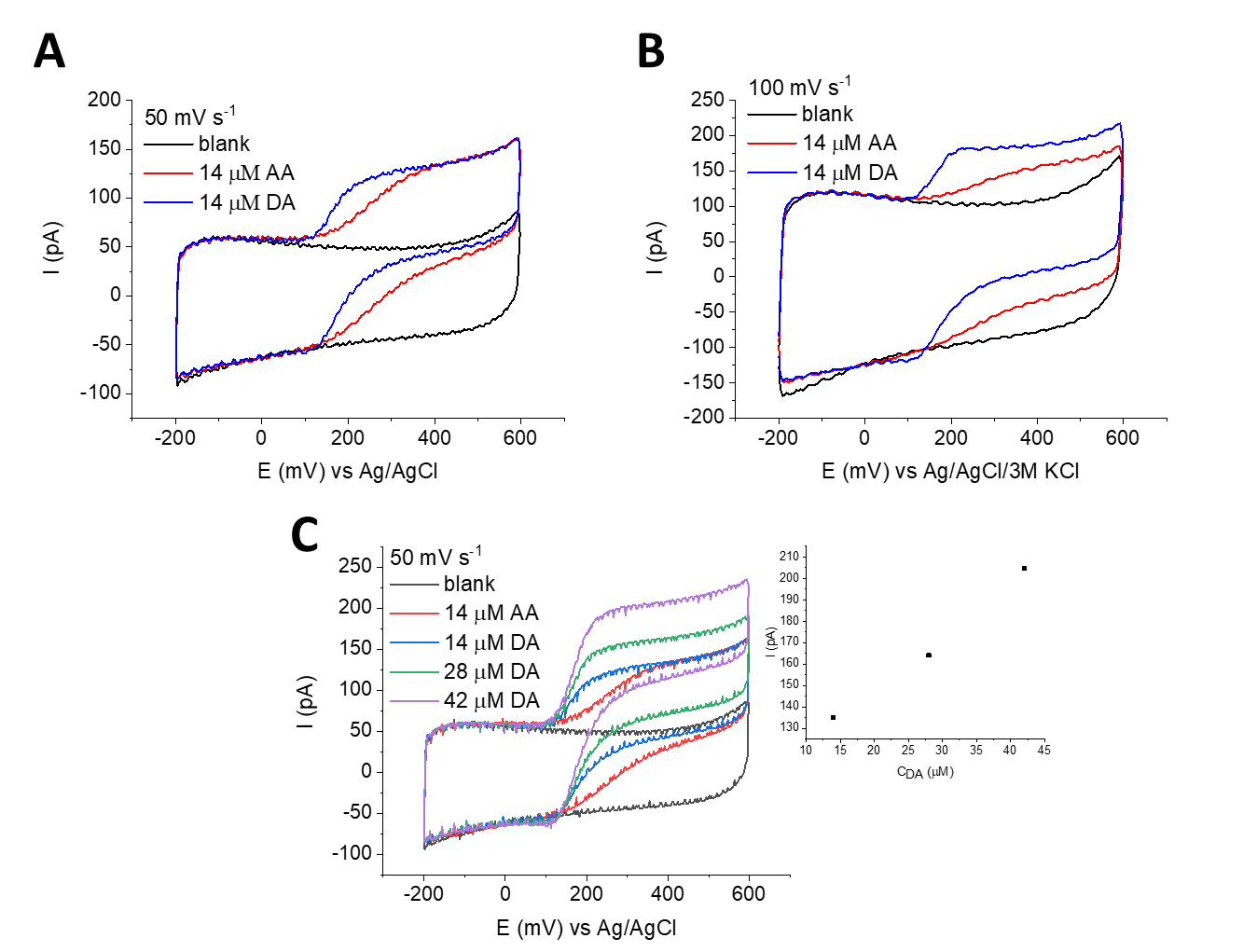


**Figure S8. Response to ascorbic acid.** CVs recorded using a needle-type gate electrode in binary mixtures containing both DA and AA at 50 mV s^-1^ (**A**) and 100 mV s^-1^ (**B**). (**C**) Voltammograms recorded at 50 mV s^-1^ in a buffered solution containing AA with increasing DA concentration. Inset: linear response of the recorded current vs DA concentration.
